# Psychoanalysis in India: A legacy in search of relevance

**DOI:** 10.1371/journal.pmen.0000335

**Published:** 2025-05-27

**Authors:** Sanjay Kumar, Mohit Kumar, Khusboo

**Affiliations:** 1 Department of Clinical Psychology, Institute of Mental Health Amritsar, India; 2 Department of Psychiatry, All India Institute of Medical Sciences, Bhopal, India; 3 Department of Psychiatry, Chirayu Medical College & Hospital, Bhopal, India; PLOS: Public Library of Science, UNITED KINGDOM OF GREAT BRITAIN AND NORTHERN IRELAND

## Psychoanalysis in India: A legacy in search of relevance

Psychoanalysis in India occupies a complex and precarious space originating through colonial-era influences, shaped by socio-political dynamics, and increasingly marginalized by biomedical psychiatry and evidence-based psychotherapies. Its historical and cultural imprints persist, yet its contemporary relevance remains under scrutiny. This opinion, aims to reach a broad audience interested in mental health practices, including professionals, service users, and advocates, with the goal of encouraging inclusive dialogue and critical reflection, explores the historical development, present status, and future potential of psychoanalysis within India’s evolving mental healthcare landscape.

Historically, psychoanalysis arrived in India in the early twentieth century through colonial transmission. Girindrasekhar Bose, known as the father of Indian psychoanalysis, initiated India’s engagement with Freudian thought [1]. His correspondence with Freud and the establishment of the Indian Psychoanalytical Society in 1922 marked critical milestones. However, psychoanalysis in India faced unique challenges from its inception, struggling to integrate into India’s diverse cultural, linguistic, and spiritual contexts. Bose’s cultural adaptations—focusing on the ego over the Oedipus complex—represented initial efforts toward contextualization [2]. Yet, psychoanalysis remained largely confined to elite circles and isolated from broader psychiatric practice.

Following India’s independence in 1947, psychoanalysis experienced considerable marginalization of these approaches within professional mental health communities, compounded by wider societal attitudes as biological psychiatry and psychopharmacology rose to prominence. Leading institutions like AIIMS, NIMHANS, and PGIMER prioritized biomedical frameworks, relegating psychoanalytic methods to peripheral status [3]. Additionally, the popularity of empirically validated therapies such as Cognitive Behavioral Therapy (CBT) and newer third-wave approaches like ACT (Acceptance and Commitment Therapy), DBT (Dialectical Behavior Therapy), and MBCT (Mindfulness-Based Cognitive Therapy) reduced the clinical appeal of psychoanalysis due to their time efficiency and measurable outcomes [3]. Compounding this was the scarcity of structured psychoanalytic training programs and qualified practitioners, further diminishing psychoanalysis’s visibility and influence.

Nevertheless, psychoanalysis endures through dedicated practitioners and institutions. Organizations such as the Indian Psychoanalytical Society (IPS), affiliated with the International Psychoanalytical Association, maintain active engagement through seminars and training programs [4]. Independent forums, notably Mumbai’s Forum for Contemporary Thinking, facilitate essential discourse and professional development. Psychoanalysis also retains academic relevance within literature, art, and cinema studies, providing valuable insights into socio-cultural phenomena [5,6].

A persistent critique is psychoanalysis’s western-centric foundations, potentially limiting its relevance within India’s collectivist and spiritually oriented culture [7]. Nonetheless, scholars like Sudhir Kakar have demonstrated psychoanalysis’s adaptability, addressing uniquely Indian contexts related to sexuality, mythology, and cultural identity [8]. Thus, psychoanalysis’s continued relevance in India hinges on deliberate localization efforts, integrating classical Indian philosophical insights such as those from the Upanishads and Abhidhamma.

For psychoanalysis to regain prominence within Indian mental health practice, it is essential to recognize its distinctive contributions. Psychoanalytic approaches offer unparalleled insights into unconscious processes, early relational experiences, and deeply entrenched emotional conflicts domains that are often insufficiently addressed by symptom-focused therapies and pharmacological treatments. Studies have shown that psychoanalysis and psychodynamic therapies are particularly effective for complex mental health conditions, such as treatment-resistant depression, borderline personality disorder, and complex post-traumatic stress disorder, where surface-level symptom management proves inadequate [9,10]. In contrast to brief interventions, psychoanalysis fosters long-term structural changes in personality and relational patterns, resulting in enduring psychological growth [11]. Furthermore, psychoanalysis emphasizes the therapeutic relationship itself as a vehicle for healing, a factor that has been increasingly recognized as crucial for successful outcomes across modalities [12]. By marginalizing psychoanalysis, communities risk losing access to depth-oriented therapeutic options that can profoundly transform lives, especially in cases where quick-fix approaches fail. Reintegrating psychoanalytic perspectives could, therefore, enrich the diversity, depth, and sustainability of mental health care in India. Additionally, several strategic directions are critical:

1. Develop indigenous psychoanalytic training programs that blend traditional theory with cultural sensitivity, tailored explicitly for Indian mental health professionals.
2. Strengthen interdisciplinary collaboration with philosophy, sociology, religious studies, and literature to enhance psychoanalytic applicability to India’s complex socio-cultural environment.
3. Create clinically practical adaptations such as brief dynamic psychotherapy and mentalization-based treatment that align effectively with India’s contemporary healthcare demands [13].
4. Expand psychoanalysis beyond clinical boundaries by engaging with cultural issues such as caste, communalism, gender dynamics, and rapid societal transformations.
5. Conduct rigorous empirical research on psychoanalytic treatment effectiveness within Indian populations, disseminating findings through prominent Indian journals to inform culturally responsive adaptations.

In conclusion, psychoanalysis in India represents a significant yet diminishing tradition overshadowed by dominant biomedical and evidence-based psychotherapies. Its future depends on meaningful cultural adaptations and innovative reimagination, addressing clinical and societal challenges relevant to contemporary Indian mental health.
